# The significance of personality disorder and traits in short-term psychodynamic and cognitive behavioral therapy for major depression

**DOI:** 10.3389/fpsyt.2026.1789466

**Published:** 2026-04-29

**Authors:** Theresa Wilberg, Randi Ulberg, Ole Klungsøyr, Anders Malkomsen, André Løvgren, Kåre Osnes, Julie Horgen Evensen, Toril Dammen, Jan Ivar Røssberg

**Affiliations:** 1 Division of Mental Health and Addiction, Oslo University Hospital, Oslo, Norway; 2 Institute of Clinical Medicine, University of Oslo, Oslo, Norway; 3 Division of Mental Health and Substance Abuse, Diakonhjemmet Hospital, Oslo, Norway

**Keywords:** depression, personality disorder, personality traits, cognitive behavioral therapy, psychodynamic therapy, psychotherapy

## Abstract

**Background:**

Research has shown inconsistent results regarding the impact of personality disorder (PD) on psychotherapy outcomes for depression. There is a scarcity of studies comparing cognitive behavioral therapy (CBT) with short-term psychodynamic therapy (STPP). This study aimed to compare outcome of STPP and CBT in depressed patients with and without PD and investigate whether PD or specific PD traits differentially affected outcome in STPP and CBT.

**Methods:**

One hundred outpatients with major depression (PD, n=28; NoPD, n=72) were randomized to STPP or CBT and followed up after 28 weeks. PD was assessed by semi-structured interview (SCID-II) which indicated mild to moderate severity of PD. Primary outcome measures were Hamilton Depression Rating Scale and Beck Depression Inventory-II, secondary outcome measures were the Global Functioning Scale, Work and Social Adjustment Scale and Generalized Anxiety Disorder 7. Statistics comprised bivariate analyses and multivariate linear regression.

**Results:**

Effect sizes for primary and secondary outcomes were large for both the PD and NoPD groups, with no significant difference in clinical status between the groups after 28 weeks. The presence of a PD diagnosis did not moderate outcome of STPP versus CBT, but the interaction between paranoid traits and treatment showed a significant effect in favor of STPP.

**Conclusions:**

The results indicate that co-occurring mild to moderate personality pathology should not be a barrier to standard psychotherapies for patients with depression. More studies are needed to investigate whether type and complexity of personality pathology differentially affect short- and longer-term outcomes of different psychotherapies for depressed patients.

## 1 Introduction

Depression represents a significant disease burden worldwide (1). Psychotherapy is documented as effective treatment for depression, comparable to pharmacotherapy in the short-term and more effective than pharmacotherapy alone in the longer-term (2–4). Yet, treatment outcome is highly variable and less than half of the patients respond sufficiently to treatment (2, 5). Accordingly, an understanding of patient-related and treatment-specific factors is important for improving treatment outcomes in depression.

Co-occurring personality disorder (PD) has been suggested as one patient factor representing a risk for poorer treatment outcome. PD is common in the general population, and in clinical samples, nearly half of patients with mood disorders have one or more PD diagnoses (6, 7). Reviews comprising a range of treatments and treatment settings, including pharmacotherapy, have found a negative impact of PD on treatment outcome (8–10). For instance, in a review of 55 studies with a broad range of methodologies, 9 found that comorbid PD more than doubled the odds of a poorer prognosis of depression compared with no PD diagnosis. The odds for poorer outcome were somewhat lower in RCTs than cohort studies, but still significant. Likewise, the review of Young (10) concluded that PD has a significant negative impact on treatment outcome in depression and underlines the importance of addressing personality pathology in treatment plans and interventions.

However, the findings that co-occurring PD is associated with poorer outcome in the treatment of depression are challenged by studies not finding significant differences in outcomes between those with and without PDs. In an early review of 50 studies Mulder (11) suggested that poorer outcome related to PD may be more pronounced in methodologically weaker studies (11). This notion is to some degree supported by more recent reviews and meta-analyses, and those restricted to RCTs (12) (13), and seems to hold for pharmacological treatments as well (14, 15). Thus, conflicting evidence regarding the impact of PD may partly depend on study methodology.

Most studies in this field are based on categorical PD diagnoses, often in terms of a single category of any specific PD diagnosis. It is increasingly acknowledged that personality pathology is dimensional phenomena ranging from mild to severe PD, as reflected in ICD-11 and the DSM-5 Alternative Model of Personality Disorder (16, 17). Thus, it may well be that the contradictory findings regarding the impact of PD reflect various severities of PD, e.g., milder forms of personality problems could affect depression treatment less than more severe disorders. This issue is clinically relevant as more knowledge may help to guide decisions as to which patients may profit from traditional depression treatments and who will need specifically tailored and more personality focused therapy.

Another source of the inconsistent findings may be potential differences in effectiveness between treatments. At this point, there is limited research as to whether PD is a moderator of outcome in some versus other types of acute phase depression treatments. With the exception of the Christchurch project in New Zealand, which found that PD and increasing PD comorbidity were associated with reduced response in interpersonal psychotherapy (IPT) in contrast to cognitive behavioral therapy (CBT) (18–20), most studies have not found that various therapies differentially affect outcome for PD in general (21–23). However, we still do not know whether different psychotherapies are particularly helpful or not regarding certain types of personality pathology. This is an under-researched area, only a few studies have directly compared different therapies with respect to various PDs or traits.

Most studies investigating the potential impact of specific PDs have compared CBT with IPT, and findings have mainly concerned cluster C PDs, i.e., avoidant, obsessive-compulsive, or dependent PD. Using data from the Second Sheffield Psychotherapy Project in UK (24), Hardy et al. (25) found that patients with cluster C PDs did significantly less well than those without PD in psychodynamic-interpersonal therapy, but not in CBT (25). Furthermore, avoidant PD traits were associated with worse outcome in IPT, but not in CBT in the Christchurch project (19). Moreover, in the NIMH Treatment of Depression Collaborative Research Program in US (26), CBT was more effective than IPT for patients with higher levels of avoidance and avoidant PD, while IPT was more effective for patients with increasing levels of obsessiveness and obsessive-compulsive PD (27). In a Canadian study (28) however, as well as in a Dutch study (23), no moderating effects of avoidant or obsessive-compulsive traits in CBT versus IPT were found.

Regarding other PD clusters, there are even fewer findings of moderating effects in CBT and IPT. In the large study of van Bronswijk et al. (23) mentioned above, which found no effect of cluster C traits or PD in general, it was patients high on cluster A features (paranoid, schizoid or schizotypal traits) that had less decrease in depression symptoms in IPT (23). Paranoid PD was the most prevalent among the cluster A PDs. In another study from the same sample aiming at a statistically constructed Personalized Advantage Index, self-reported paranoid symptoms were among the significant variables predicting a better response to CBT compared to IPT (29). Finally, in the Christchurch study, both paranoid, schizotypal and borderline symptoms were associated with poorer response in IPT in univariate analyses in addition to avoidant symptoms, but only avoidant and to a lesser degree schizoid traits contributed significantly in multivariate analyses (19).

Psychodynamic therapy (PDT) is a commonly applied bona fide therapy for depression, with generally equal efficacy as CBT (5, 30). Yet, in only one project has CBT been compared with PDT to investigate the potential moderating role of PD or PD traits. In a large multi-center study in Amsterdam patients were randomized to 16 sessions of short-term psychodynamic supportive psychotherapy (SPSP, n=104) or CBT (n=92) (21). Both patients with and without PDs improved with large effect sizes (ESs), but patients with PD had poorer depression outcomes. There was, however, no effect of treatment conditions between those with and without PD. Neither were there any interaction effects of avoidant or obsessive traits (31). Yet, a significant limitation in the project was that PD was assessed only by a screening questionnaire known to grossly overestimate the prevalence of PD.

In sum, research regarding the role of PD in the outcome of psychotherapy for depression has shown inconsistent results. Many questions remain concerning the significance of the extent of personality pathology for treatment response and whether the negative impact of PD mainly concerns more severe disorders. If patients with less severe PD may profit equally well as those without PD, this may have implications for the organization of services and PD patients’ access to standard depression therapy. Moreover, we need to know whether certain types of PD or PD traits are associated with better response to some versus other therapies. Such knowledge could be a step towards more personalized therapy. Earlier studies of such moderating effects have mainly investigated CBT and IPT, and more studies comparing CBT with short-term PDT are needed.

## 2 The present study

The present study is part of the MOP study (Mechanisms of Change in Psychotherapy), an RCT of treatment of depression focusing on moderators and mediators of change in CBT and short-term psychodynamic therapy (STPP) (32). A previous paper from MOP documented large within effect sizes in outcome of depression with no significant differences between the treatments (30). There was, however, considerable heterogeneity of outcomes. According to standard definitions on depression measures 36% – 54% of the patients were treatment responders in terms of a minimum of 50% symptom reduction, and around one third of the patients’ experienced remission.

In the present study, we aimed to investigate the clinical outcome in patients with co-occurring PD as compared to patients with no PD diagnosis, as well as the potential influence of the extent of personality pathology. We further aimed to investigate potential differences in outcomes between treatments and whether certain forms of personality pathology influenced outcomes differently in CBT and STPP. The primary outcome was depression as assessed by Hamilton Depression Rating Scale (HDRS, 33) and Beck Depression Inventory-II (BDI-II, 34). Secondary outcomes were psychosocial functioning and anxiety as assessed by the Global Functioning Scale (GFS, 35), the Work and Social Adjustment Scale (WSAS, 36), and Generalized Anxiety Disorder 7 (GAD-7, 37).

## 3 Research questions

**Figure 1:**
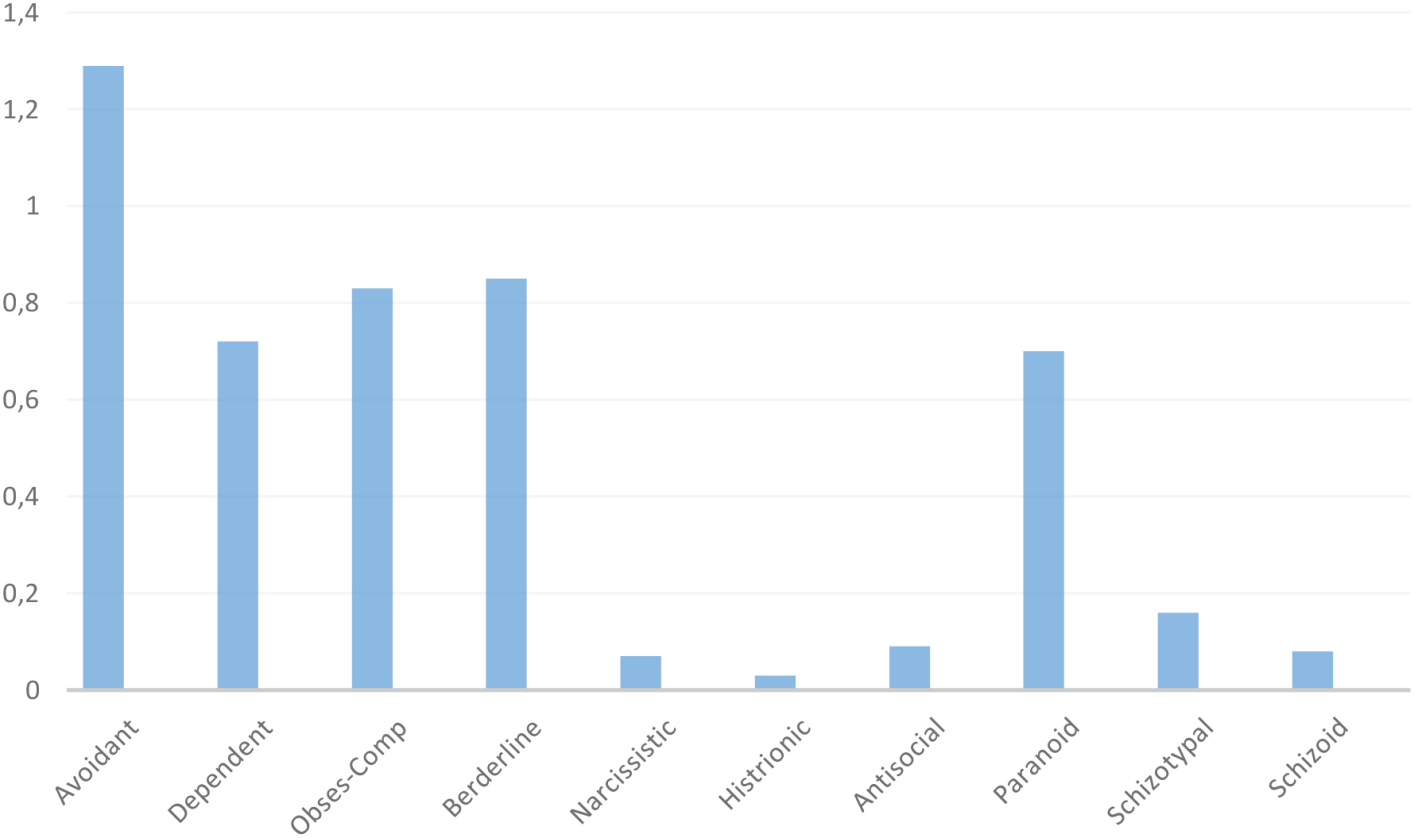
The prevalence of PD traits (SCID-II criteria) in the total sample.

1. How did patients with depression and comorbid PD benefit from CBT and STPP compared with those without PD? Within the present context of an RCT and mainly comprising patients with mild to moderate personality pathology, our first hypothesis was that the outcome of patients with a co-occurring PD diagnosis would not differ from depressed patients without PD. However, when applying a dimensional approach to personality pathology, our second hypothesis was that a greater extent of personality pathology, as reflected in a higher number of PD traits, would be associated with less improvement.
2. Was PD a moderator of outcome in CBT and STPP, i.e., were there differences in response between patients with or without PD in CBT as compared with STPP? We expected that PD would not influence outcome differently in CBT and STPP, neither as a single diagnostic category nor as total number of PD traits.
3. Did certain PD traits differentially influence the outcome of CBT and STPP? Earlier studies have mainly compared CBT and IPT, focusing mostly on cluster C traits and less on other PD clusters. The distribution of traits is shown in **Figure 1**. The MOP protocol, based on research at the time, stated the following hypotheses regarding specific traits; patients with more avoidant personality traits and more paranoid symptoms/traits would report better outcomes of CBT than STPP (32). Although previous findings are mixed, in the present study we stuck to our pre-registered protocol hypotheses and expected that avoidant and paranoid personality traits would be associated with better response in CBT than STPP.

## 4 Methods

### 4.1 Setting and design

The present study comprises patients included in the MOP study, an RCT of short-term treatment of depression conducted at two public psychiatric outpatient clinics in Oslo, Norway (32). The MOP study was approved by The Regional Committee for Medical and Health Research Ethics, Southeast Norway (REK 2016/340) and registered in Clinical Trial go. Identifier: NCT03022071. The participants provided their written informed consent to participate in the study.

The inclusion criterion was major depressive disorder (MDD), while exclusion criteria were psychotic disorders, bipolar disorder type I, current alcohol and/or substance dependence disorders, developmental disorders, intellectual disability, traumatic brain injury, and current or past neurological illness. The patients were randomized to either CBT (one weekly session over 16 weeks and three monthly booster sessions) or STPP (one weekly session over 28 weeks), thus treated over 28 weeks in both conditions. The CBT was based on “Cognitive Therapy of Depression” by Aaron Beck (38), and the STPP applied principles from “Long-term psychodynamic psychotherapy” by Glen O. Gabbard (39) adapted to the short-term time frame as described by Cregeen et al. (40). Further details of the treatments are described in a previous paper (30), which also showed that the CBT and STPP differed significantly when using the Comparative Psychotherapy Process Scale ratings of therapy sessions (41). The present study is based on evaluations at baseline and after 28 weeks, which is at the end of therapy for most patients.

### 4.2 Assessments

#### 4.2.1 Diagnostics

Personality disorder (PD) diagnoses were assessed by the Structured Clinical Interview for DSM-IV Axis II Personality Disorders (SCID-II, 42). Symptom disorders were assessed using the Mini International Neuropsychiatric Interview 6.0.0 (MINI, 43). The diagnostic evaluations were performed at baseline by research team clinicians not taking part in the study treatments. In the present study, the number of fulfilled SCID-II criteria was applied as a proxy for PD severity. The reliability of this severity index, estimated by ICC (2.1) based on scores from six raters scoring 11 patients, was 0.99 (CI: 0.97-1.00).

#### 4.2.2 Primary outcomes

Levels of depression assessed by both observer-rated and self-report methods constitute the primary outcomes in the study. Hamilton Depression Rating Scale (HDRS, 33) is an observer-rated measure composed of 17 items, each rated on a scale from 0 to 4 or 0 to 2, covering a range of depression symptoms experienced during the past week. Higher scores indicate more severe depression. The total sum scores are typically categorized as follows: 0-7 (normal), 8-13 (mild depression), 14-18 (moderate depression), 19-22 (severe depression), and ≥ 23 (very severe depression). HDRS was rated at baseline and after 28 weeks by research team clinicians not taking part in the treatment and blind to the treatment allocation. The reliability of HDRS was estimated by ICC (2.K) (44) based on four raters scoring 10 patients. The reliability coefficients were 0.97 (CI: 0.92–0.99) and 0.96 (CI:0.86–0.99) for relative decision and absolute decision, respectively. The Beck Depression Inventory-II (BDI-II, 34) is a 21-item self-report measuring the severity of various depressive symptoms during the past week. Each item is rated on a 0 to 3 scale, with higher scores indicating more severe depression. Established cut-offs for the total sum score are: 0-13 (minimal depression), 14-19 (mild depression), 20-28 (moderate depression), and 29-63 (severe depression). Beck-II has demonstrated good psychometric properties (45).

#### 4.2.3 Secondary outcomes

Psychosocial functioning was assessed by observer rated as well as self-report measures.

The observer rated *Global Functioning Scale* (GFS, 35) is a revised version of the Global Assessment of Functioning Scale (APA 1994) that can be split into a function score (GFS-F) and symptom score (GFS-S). GFS scores range from 1-100 with lower scores representing more symptom severity and functional impairment, i.e., GFS-F < 50 representing increasingly severe impairment, GFS-F 50 – 60 moderate impairment, and GFS-F > 60 indicating mild to no impairment. GFS-F and GFS-S with reference to the past week were evaluated at baseline and after 28 weeks by the research team clinicians. Reliability was estimated by ICC (2.K) (T. K. Koo & M. Y. Li, 2016) based on four raters scoring 11 patients. For GFS-F, the reliability coefficient for relative decision was 0.83 (CI: 0.57–0.95) and 0.82 (CI: 0.57–0.95) for absolute decision. For GFS-S, the reliability coefficients were 0.92 (CI: 0.80–0.98) and 0.90 (CI: 0.74–0.97), respectively. The Work and Social Adjustment Scale (WSAS, 36) is a five-item self-report questionnaire measuring impairment in work, social leisure activities, private leisure activities, home management, and close relationships over the past month. Each item is scored on a scale ranging from 0 (not at all) to 8 (severely). Total sum scores above 30 indicate severe disability, scores between 15 and 30 moderate impairment, and scores below 15 can be regarded as mild impairment or disability. WSAS has been shown to have high internal reliability, and is sensitive to treatment effects (46). In addition the patients’ level of anxiety was assessed by the Generalized Anxiety Disorder 7 (GAD-7, 37), a 7-item self-report measuring common symptoms of anxiety like excessive worry, restlessness, and difficulty concentrating. Patients rate the frequency of each symptom over the past two weeks on a scale ranging from 0 (not at all) to 3 (nearly every day). Total sum scores range from 0 to 21, with the established cut-offs: 0-4 (minimal anxiety), 5-9 (mild anxiety), 10-14 (moderate anxiety), and 15-21 (severe anxiety). GAD-7 has shown good psychometric properties and validity (47, 48). The self-report questionnaires BDI-II, WSAS and GAD-7 were filled in on several occasions during treatment. To apply similar statistical methods for all outcome measures, only the baseline and 28 weeks assessments were used in the present study.

#### 4.2.4 Participants

A total of 100 patients were included, mean age 31.2 (SD = 8.7), 59% females and 39% married or cohabiting. Sixty-two percent of the total sample had previous experience with psychotherapy, 7% had previously been hospitalized, and 42% used antidepressive medication at baseline. There were no significant differences in sociodemographic or clinical variables at baseline between patients randomized to CBT (n=50) or STPP (n=50) (30).

Among the 100 patients 28 patients had a PD diagnosis (CBT: n=13; STPP: n=15). All patients had only one PD diagnosis, except one patient with co-occurring avoidant and dependent PD. The distribution of PD diagnoses was: avoidant 15%, paranoid 5%, dependent 2%, obsessive-compulsive 1%, PD NOS 6%. Mean number of fulfilled PD criteria in the total sample was 4.8 (SD = 4.3), median 4.0 (range 0 – 18). There were no statistically significant differences between patients without PD diagnosis (the NoPD group) and the PD group regarding sociodemographic and diagnostic variables or previous treatment at baseline (**Table 1**), other than the PD group having significantly more fulfilled SCID-II criteria (NoPD: 2.7, SD = 2.4 vs PD:10.2, SD = 3.3; p<.001), as well as a trend that the PD group had more symptom disorders (NoPD: 1.9, SD = .09 vs PD: 2.4, SD = 1.2; p=.062, Mann-Whitney test). Forty-four percent of patients in the NoPD group and 36% in the PD group used antidepressive medication at baseline (p=.427). At follow-up respectively 46% and 42% in the NoPD and PD group used antidepressants (p=.744).The distribution of patients using antidepressants did not differ significantly between the treatments (baseline: CBT = 48%, STPP = 36%; p= .224 and follow-up: CBT = 46%, STPP = 43%; p=.765). The NoPD and PD group did not differ significantly in number of sessions attended (NoPD, 18.3, SD = 7.9; PD, 19.8, SD = 6.0).

**Table 1:** Socio-demographic and clinical characteristics at baseline among patients without PD diagnosis (n=72) and those with comorbid PD (n=28).

Variable	No PD %	Comorbid PD %	Sig. dif. p
Age, mean (sd)	31.4 (9.0)	30.5 (7.7)	.645
Female	60	57	.825
Married/cohabiting	40	36	.820
No. years of education after junior high school, mean, (sd)	6.0 (2.7)	6.2 (3.0)	.741
No. months in at least 50% work/study last year, mean (sd)	7.9 (4.7)	9.2 (3.6)	.218
Previous psychotherapy	61	64	.822
Previous hospitalization	7	7	.100
Antidepressive medication	44	36	. 502
No. of symptom disorders, mean (sd)	1.9 (.9)	2.4 (1.2)	.062^c^
MDD^a^ Single episode Recurrent episode	11 88	11 89	.100
Anxiety disorders	53	68	.186
Substance use disorders	6	7	.671
No. PD criteria^b^	2.7 (2.4)	10.2 (3.3)	<.001
No. attended treatment sessions, mean (sd)	18.3 (7.9)	19.8 (6.9)	.381

a Major Depressive Disorder; ^b^ Number of fulfilled SCID-II criteria; ^c^ Mann-Whitney test.

81 patients attended the follow-up assessment 28 weeks after treatment start, of whom 30% (n=24) had a PD diagnosis at baseline. Patients who did not attend the follow-up had fewer years of education after junior high school than those attending (4.9, SD = 3.0 vs 6.3, SD = 2.6; p=.045) and attended fewer treatment sessions (11.3, SD = 7.3 vs 20.41, SD = 6.6; p<.001). The groups did not differ regarding gender, marital status, previous treatment, antidepressive medication, number of symptom disorders, or number of PD criteria, and there were no indications that those who did not attend the follow-up had more impaired psychosocial functioning or higher symptom burden at baseline. Among patients who attended the follow-up there was no significant difference in number of treatment sessions attended between the NoPD and the PD group (NoPD, 20.1, SD = 6.8; PD, 21.0, SD = 6.1).

#### 4.2.5 Statistics

All analyses used SPSS Statistics for Windows, Version 30. Bivariate analyses included Independent t-test, Mann-Whitney U-test, Pearson Chi-Square and Fisher’s Exact Test. Effect sizes (ES) for outcomes in patients with and without co-occurring PD was calculated by Cohen’s d with pooled SDs correcting for uneven sample sizes. The magnitude of the ES may be interpreted using Cohen’s convention as small (0.2), medium (0.5) and large (0.8) (49). Treatment response in depression was defined as a reduction of ≥ 50% in HDRS and BDI-II respectively, and remission was specified as levels of HDRS ≤ 7 and BDI-II ≤ 9. Linear regression analyses, enter method, were applied to investigate if PD or PD traits predicted or moderated outcomes in CBT and STPP. Change scores in outcome measures were entered as dependent variables in separate analyses, controlling for baseline levels. Then, to investigate the impact of the extent of personality pathology the total number of PD criteria was added as independent variable. To answer the second research question, we entered PD or total PD criteria respectively as independent variables into the regression models and then added treatment group and the interaction term treatment x PD/PD criteria. The question of whether avoidant and paranoid PD traits differentially influenced outcome in CBT and STPP was investigated by adding independent variables in the following order: number of PD criteria other than avoidant or paranoid respectively, avoidant or paranoid criteria, treatment group, and finally treatment X avoidant/paranoid criteria in the final step. An alpha level of p<.05 was applied for statistical significance.

## 5 Results

Nine patients in the NoPD group and two patients in the PD group dropped out of treatment, defining dropout as attending fewer than eight sessions. The difference was not statistically significant.

### 5.1 Primary outcomes

For the primary outcomes HDRS and BDI-II the pre-post ESs were large for both patients with and without PD diagnosis (**Table 2**). The PD group had a significantly higher level of HDRS at baseline (p<.001) and significantly greater change in HDRS compared to the NoPD group (p=.032). Baseline BDI-II and BDI-II change did not differ between the groups. After 28 weeks there were no statistically significant differences in HDRS or BDI-II between the groups. The rates of treatment response did not differ significantly between the groups (HDRS: NoPD, 39% vs PD, 50%, p=.342) (BDI-II: NoPD, 57% vs PD, 42%, p=.204). Neither did the groups differ significantly regarding remission on HDRS (NoPD, 33% vs PD, 29%, p=.714) or BDI-II (NoPD, 38% vs PD, 29%, p=.474).

**Table 2:** Primary and secondary outcomes, i.e., depression, psychosocial functioning and anxiety in patients without PD diagnosis and those with comorbid PD.

	No PD			Comorbid PD			
Outcome measure	Mean (SD)	N	ES^*^	Mean (SD)	N	ES^*^	Sig. dif. p
Primary outcomes							
HDRS^a^ pre	16.88 (4.87)	72		21.21 (6.29)	28		<.001
week 28	10.42 (5.30)	57		10.58 (4.64)	24		.897
HDRS pre-post dif	6.79 (6.41)	57	1.28	10.21 (6.47)	24	1.90	.032
BDI-II^b^ pre	26.76 (7.32)	72		29.50 (8.01)	28		.105
week 28	14.98 (10.0)	56		15.96 (10.26)	24		.692
BDI-II pre-post dif	11.39 (11.97)	56	1.36	12.46 (8.11)	24	1.49	.692
Secondary outcomes							
GFS-F^c^ pre	56.96 (5.61)	72		54.75 (5.29)	28		.038
week 28	68.09 (9.22)	57		67.00 (9.63)	24		.317
GFS-F pre-post dif	-11.79 (9.29)	57	1.50	-12.17 (8.51)	24	1.61	.865
GFS-S^d^ pre	53.39 (4.12)	72		51.04 (3.74)	28		.005
week 28	63.00 (8.01)	57		61.96 (8.80)	24		.303
GFS-S pre-post dif	-10.05 (8.61)	57	1.56	-11.17 (9.47)	24	1.66	.607
WSAS^e^-sum pre	24.81 (6.11)	72		28.00 (7.35)	28		.029
week 28	16.23 (9.48)	56		17.92 (10.57)	24		.484
WSAS pre-post dif	8, 20 (9.42)	56	1.11	9.29 (10.36)	24	1.12	.645
GAD7^f^-sum pre	12.85 (4.61)	72		14.64 (3.58)	28		.060
week 28	9.13 (5.32)	55		10.13 (4.71)	24		.430
GAD7 pre-post dif	3.71 (5.23)	55	.75	4.21 (5.30)	24	1.09	.698

*Effect size, Cohen’s d with pooled SDs correcting for uneven sample sizes; ^a^Hamilton Depression Rating Scale; ^b^Beck Depression Inventory-II; ^c^Global Functioning Scale, Function Score; ^d^ Global Functioning Scale, Symptom Score; ^e^Work and Social Adjustment Scale; ^f^Generalized Anxiety Disorder 7.

In the regression analyses, higher HDRS at baseline contributed significantly to larger HDRS change (β=.600, p<.001) explaining 44% of the variance (ΔF=61.592, p<.001) in HDRS change (**Table 3**). A higher number of PD criteria contributed to further HDSR change (β=.194, p=.029), but accounted for only 3% additional variance, i.e., 3% (ΔF=.4.973, p =.029). Higher baseline BDI-II values contributed significantly to more BDI-II change (β=.448, p<:001) explaining 21% of the variance (ΔF=20.505, p<.001). The number of PD criteria gave no further significant contribution (β=.083, p=.417).

**Table 3:** Multiple regression analyses with change scores for primary and secondary outcomes as dependent variables.

Dependent	Independent	B	SE	β	p	ΔR^2^	Total R^2^ adjusted
HDRS^a^ change (n=81)	HDRS pre No. PD criteria^g^	.703 .289	.102 .129	.600 .194	**<.001** **.029**	.438 .034	.458
BDI-II^b^ change (n=80)	BDI pre No. PD criteria	.657 .203	.149 .249	.448 .083	**<.001** .417	.208 .007	.195
GFS-F^c^ change (n=81)	GAF-f pre No. PD criteria	-.403 .525	.171 .216	-.248 .257	**.021** **.017**	.066 .066	.110
GFS-S^d^ change (n=81)	GAF-s pre No. PD criteria	-.822 .390	.261 .210	-.331 .195	**.002** .067	.136 .037	.151
WSAS^e^ change (n=80)	WSAS pre No. PD criteria	.472 .205	.154 .233	.327 .094	**.003** .382	.108 .009	.094
GAD7^f^ change (n=79)	GAD7 pre No. PD criteria	.498 .107	.130 .124	.408 .092	**<.001** .390	.184 .008	.171

Change refers to pre scores minus post scores in primary and secondary outcomes, except for GFS where change refers to post scores minus pre scores.

a Hamilton Depression Rating Scale; ^b^ Beck Depression Inventory-II; ^c^ Global Functioning Scale, Function Score; ^d^ Global Functioning Scale, Symptom Score; ^e^ Work and Social Adjustment Scale; ^f^Generalized Anxiety Disorder 7; ^g^ Number of fulfilled SCID-II criteria.

P values < .05 are indicated in bold.

### 5.2 Secondary outcomes

The pre-post ESs were large for the secondary outcomes for both patients with and without PD, except for a medium effect size for GAD7 in the NoPD group (**Table 2**). At baseline the PD group had a significantly higher level of WSAS compared to the NoPD group (NoPD, 24.81 ± 6.11 vs PD, 28.00 ± 7.35; p=.029), as well as lower levels of GFS-S (NoPD, 53.39 ± 4.12 vs PD, 51.04 ± 3.74; p=.005) and GFS-F (NoPD, 56.96 ± 5.61 vs PD, 54.75 ± 5.29; p=.038). However, there were no significant differences between the groups in any of the secondary outcomes after 28 weeks and no significant differences in change scores.

In the regression analyses, lower levels of GFS-F at baseline were significantly associated with more change in GFS-F (β=-.248, p=.021) explaining 7% of the variance (ΔF=5.596, p=020). A higher number of PD criteria contributed significantly to larger change in GFS-F (β=.257, p=.017), explaining additionally 7% of the variance (ΔF=5.926, p=.017) (**Table 3**). Regarding GFS-S a lower baseline level was associated with more change (β=-.331, p=.002) accounting for 14% of the variance (ΔF=12.396, p<.001), but number of PD criteria did not add significantly to further GFS-S change. Also, more self-reported problems with psychosocial adjustment as assessed by WSAS were associated with more positive change (β=.327, p=.003), explaining 11% of the variance in WSAS change (ΔF=9.461, p=.003), whereas number of PD criteria had no additional significance. Likewise, higher baseline levels of GAD7 contributed to larger change in GAD7 (β=.408, p<.001) accounting for 18% of the variance (ΔF=17.385, p<.001) with no further contribution by PD criteria.

### 5.3 Personality disorder as a potential moderator of outcome in CBT and STPP

**Figure 2:**
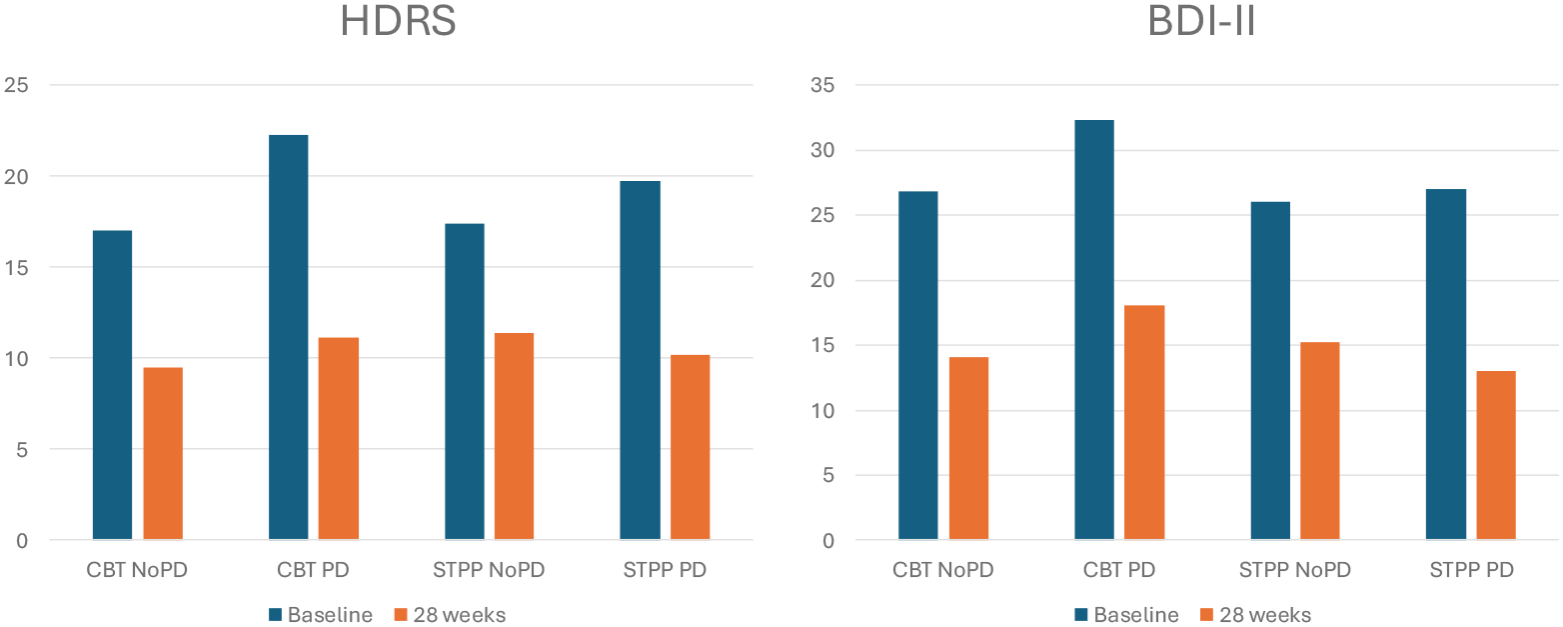
Levels of HDRS and BDI-II at baseline and after 28 weeks in the NoPD and PD group in CBT and STPP among patients who attended the follow-up (n=81). HDRS, Hamilton Depression Rating Scale; BDI-II, Beck Depression Inventory.

Among the 81 patients who attended the follow-up, the proportion of patients with PD did not differ significantly between the treatment groups (CBT: n=10, 26%; STPP: n=14, 33%; p=.449). The average number of PD traits in the CBT group was 5.0 ± 4.37 (median 4, range 0 – 17) and the average number of traits in the STPP group was 4.71 ± 4.51 (median 3.5, range 0 – 18) with no significant difference in distribution between treatments (p=.686). Baseline and 28 week levels of HDRS and BDI are shown in **Figure 2**. Neither treatment nor the interaction terms treatment x PD/PD criteria contributed significantly to change in HDRS, BDI-II or any of the secondary outcomes, explaining between zero and 2% of the variance in change scores. In line with our hypothesis, the results indicated no moderating effect of a PD diagnosis or total amount of PD criteria regarding outcomes in CBT versus STPP. .

### 5.4 Effect of specific PD traits

Of those 81 patients who attended the follow-up, 38 patients (47%) had at least one avoidant trait with an average of 2.97 ± 1.85 traits (median 2, range 1 - 7), and no significant difference in distribution between treatments (p=.469). The three most common avoidant traits were: Is preoccupied with being criticized or rejected in social situations (N = 23), Is unwilling to get involved with people unless certain of being liked (N = 20), and Views self as socially inept, personally unappealing, or inferior to others (N = 16). The regression analyses showed no significant interactions between the number of avoidant traits and treatment group regarding change in any primary or secondary outcomes (**Table 4**).

**Table 4:** The influence of avoidant and paranoid PD traits in multiple regression analyses with change scores for primary and secondary outcomes as dependent variables.

PD traits	Change	Independent	B	SE	β	p	ΔR^2^
Avoidant traits	HDRS^a^ change (n=81)	HDRS pre Other than avoidant traits Avoidant traits Treatment Avoidant x Treatment	.686 .554 -.376 -.971 .155	.101 .179 .473 1.306 .575	.585 .285 -.112 -.074 .040	**<.001** **.003** .429 .460 .789	.438 .060 .007 .003 .000
	BDI-II^b^ change (n=80)	BDI pre Other than avoidant traits Avoidant traits Treatment Avoidant x Treatment	.678 .476 -.413 .901 -.030	.153 .354 .981 2.728 1.195	.462 .150 -.074 .042 -.005	**<.001** .183 .675 .742 .980	.208 .015 .004 .001 .000
	GFS-F^c^ change (n=81)	GAF-f pre Other than avoidant traits Avoidant traits Treatment Avoidant x Treatment	-.446 1.006 -.550 .502 -.048	.170 .302 .829 2.303 1.017	-.275 .377 -.119 .028 -.009	**.011** **.001** .509 .828 .962	.066 .113 .013 .001 .000
	GFS-S^d^ change (n=81)	GAF-s pre Other than avoidant traits Avoidant traits Treatment Avoidant x Treatment	-.815 .917 -1.006 -1.122 .386	.260 .291 .788 2.170 .973	-.328 .351 -.223 -.064 .075	**.002** **.002** .206 .607 .693	.136 .083 .026 .001 .002
	WSAS^e^ change (n=80)	WSAS pre Other than avoidant traits Avoidant traits Treatment Avoidant x Treatment	.484 .468 -.176 3.851 -.331	.156 .328 .897 2.517 1.103	.336 .166 -.036 .201 -.059	**.003** .158 .845 .130 .765	.108 .016 .002 .031 .001
	GAD7^f^ change (n=79)	GAD7 pre Other than avoidant traits Avoidant traits Treatment Avoidant x Treatment	.490 .204 -.049 -.316 -.153	.131 .175 .470 1.330 .578	.401 .135 -.018 -.030 -.051	**<.001** .247 .918 .813 .792	.184 .016 .004 .002 .001
Paranoid traits	HDRS change (n=81)	HDRS pre Other than paranoid traits Paranoid traits Treatment Paranoid x Treatment	.718 .140 -.379 -2.598 2.128	.100 .156 .790 1.232 .989	.612 .082 -.064 -.199 .305	**<.001** .374 .633 **.038** **.035**	.438 .025 .016 .009 .030
	BDI-II change (n=80)	BDI pre Other than paranoid traits Paranoid traits Treatment Paranoid x Treatment	.676 .158 -2.682 -2.895 4.795	.150 .305 1.537 2.544 1.972	.461 .057 -.276 -.133 .408	**<.001** .606 .085 .259 **.017**	.208 .007 .000 .000 .058
	GFS-F change (n=81)	GFS-F pre Other than paranoid traits Paranoid traits Treatment Paranoid x Treatment	-.340 .322 -.753 -2.692 3.582	.176 .270 1.449 2.210 1.811	-.209 .138 -.093 -.150 .375	.057 .236 .605 .227 .052	.066 .051 .025 .001 .043
	GFS-S change (n=81)	GFS-S pre Other than paranoid traits Paranoid traits Treatment Paranoid x Treatment	-.780 .026 .576 -3.449 2.760	.256 .253 1.316 2.064 1.649	-.314 .011 .072 -.196 .295	**.003** .918 .663 .099 .098	.136 .019 .059 .009 .028
	WSAS change (n=80)	WSAS pre Other than paranoid traits Paranoid traits Treatment Paranoid x Treatment	.464 .117 -1.587 .402 3.713	.152 .281 1.437 2.383 1.843	.322 .047 -.184 .021 .357	**.003** .679 .273 .866 **.048**	.108 .007 .004 .023 .045
	GAD7 change (n=79)	GAD7 pre Other than paranoid traits Paranoid traits Treatment Paranoid x Treatment	.481 .017 -.358 -1.782 1.508	.131 .152 .759 1.261 .976	.393 .013 -.077 -.172 .269	**<.001** .912 .639 .162 .127	.184 .004 .010 .005 .025

Change refers to pre-scores minus post-scores in primary and secondary outcomes, except for GFS where change refers to post scores minus pre-scores. Treatment reference category: cognitive behavioral therapy=0, short-term psychodynamic therapy=1.

a Hamilton Depression Rating Scale; ^b^ Beck Depression Inventory-II; ^c^ Global Functioning Scale, Function Score; ^d^ Global Functioning Scale, Symptom Score; ^e^ Work and Social Adjustment Scale; ^f^ Generalized Anxiety Disorder 7.

P values < .05 are indicated in bold.

Thirty-one patients (38%) of those who attended the followed-up had at least one paranoid trait with an average of 1.71 ± 1.19 traits (median 1, range 1-5) with no significant difference in distribution between treatments (p=.604). The three most common paranoid criteria met were: Is reluctant to confide in others because of unwarranted fear that the information will be used maliciously against him or her (N = 11), Persistently bears grudges, i.e., is unforgiving of insults, injuries, or slights (N = 10), and Perceives attacks on his or her character or reputation that are not apparent to others and is quick to react angrily or to counterattack (N = 10). Unexpectedly, there were significant interactions between paranoid traits and treatment group in favor of STPP on the primary outcomes HDRS and BDI-II, as well as the secondary outcome WSAS, and a close to statistically significant interaction for GFS-F (p=.052) (**Table 4**). The interaction between paranoid traits and treatment explained between 3% and 6% of additional outcome variance.

In addition to testing our preregistered hypotheses we explored possible moderator effects on the primary outcomes of the three other most frequent types of PD traits in the sample, i.e., borderline, obsessive-compulsive and dependent traits, applying the same regression modeling as above. There were no significant interactions between these specific traits and change in primary outcomes, thus no indication that these traits differentially affected depression outcome in CBT and STPP.

## 6 Discussion

The present study investigated the significance of PD for the outcome of CBT and STPP for MDD within the context of an RCT in outpatient psychiatric settings. Our first main finding was that the presence of a PD diagnosis to a limited degree affected clinical outcome. Contrary to expectations, an increasing number of PD traits was associated with more improvement in observer-rated depression and functional impairment. In line with many studies, patients with PD had initially higher levels of depression and symptomatic distress, and more problems with psychosocial functioning (12, 21, 23). The PD group did, however, not differ significantly from the NoPD group after 28 weeks. Thus, the results stand in contrast to studies finding that despite similar improvements, PD patients often end up more depressed after treatment, and with lower remission rates, due to their more severe initial symptom levels (12, 22, 50, 51). The present results may reflect that the personality pathology in this sample was mainly of mild to moderate severity. Only one patient had more than one PD diagnosis, indicating that patients with more extensive and complex PDs were not represented. Moreover, the prevalence of PD diagnoses was lower than expected in clinical samples (6), and no patients had borderline PD. Patients with borderline or other more severe personality pathology had most likely been referred to group therapy or specialized PD treatment programs, which were also part of the treatments offered at these outpatient clinics. The findings are promising, suggesting that patients with MDD and mild to moderate co-occurring personality pathology may be treated with standard depression treatment in a general psychiatric outpatient setting, and with low risk of treatment dropout.

Baseline levels of depression and psychosocial problems accounted for the largest part of outcome variance. Yet, when controlling for initial severity, having more PD traits contributed to additional improvement in two of five outcomes. The additional explained outcome variance was small though, and the clinical significance may be questioned. Nevertheless, for many patients, depression occurs as part of negative self-esteem, maladaptive response patterns, or relational difficulties. Such problems, indicative of various personality difficulties, are therefore natural themes in psychotherapy and may constitute meaningful material to be worked with in the treatments. These results are relevant to mental health services. If mild to moderate personality pathology is not an obstacle to standard psychotherapy for depression, this knowledge may help decisions regarding the allocation of treatment resources. With limited resources, more resources may be allocated to the treatment of severely personality disordered patients in need of more intensive or specialized personality focused treatment for their depression.

It’s important to note, however, that the present study comprised short-term psychotherapies. A significant challenge in short-term treatment of depression is the high rates of depression relapse, and residual symptoms, impaired global functioning, and anxiety are among the risk factors (52–55). In line with most studies the majority of patients in the present study did not obtain full remission of their depression (2), and many patients still had mild to moderate psychosocial impairments and anxiety levels, making them vulnerable to relapse. PD is typically characterized by long-lasting problems with self- and interpersonal functioning, including emotional dysregulation (17, 56). Even if the PD group did not have poorer response than the NoPD group, we don’t know if these patients still had poorer coping skills or more interpersonal difficulties which could impact their future social functioning and increase the risk of increase or relapse of depression (57). Follow-up studies investigating the longer-term clinical course are needed to provide a more complete picture of the significance of PD for the outcome of short-term depression treatment.

The next main finding supported our hypothesis that neither the presence of a PD diagnosis nor the number of PD traits differentially affected the outcome of CBT and STPP. The lack of a moderating effect of PD diagnosis is in accordance with the only previous study comparing CBT and PDT (21), as well as those comparing CBT and IPT (23), except the Christchurch project, where a larger number of PD traits had a negative effect on depression outcome in IPT (19). Thus, a PD diagnosis per se seems not sufficiently informative to guide selection among various types of short-term psychotherapies. Knowledge of the patients’ specific PD traits could be more helpful in this respect.

The present study adds to the scarce research on the significance of PD traits in different treatments. Contrary to our pre-registered protocol hypotheses avoidant and paranoid traits were not associated with better outcomes in CBT than STPP. On the contrary, whereas avoidant traits did not differentially affect outcomes, in line with some other studies (23, 28, 31), paranoid traits were associated with better outcomes in STPP. At this point, we can only speculate on the unexpected result regarding paranoid traits. Previous studies of potential differential influence of cluster A PDs concern IPT and have inconsistent findings (19, 22, 23, 29). STPP as applied in MOP bear some resemblance with IPT, but there are also significant differences between the therapies (58). While current interpersonal relationships are important topics in both therapies, IPT mainly centers on the patient’s current interactions with the external environment. STPP has a broader focus, which includes how early experiences may influence current interpersonal patterns and emotional struggles, focusing on difficult emotions, defense mechanisms and other unconscious material. Moreover, whereas IPT is a more structured approach with an active therapist using explicit tools, e.g., role-playing, an STPP therapist is less active, combining exploration and interpretation of sensitive topics, including the relationship to the therapist (30). It could be that patients with pervasive relational distrust, resentment, and a tendency to interpret others’ motives as malevolent have profited from the more open and explorative approach in STPP, which also encourages reflections on the therapeutic relationship. Yet, the speculative nature of this hypothesis is underscored by the contrary suggestion by von Bronswijk et al., i.e., that these patients may benefit from more structured treatments, based on their finding that patients high on cluster A traits fared better in CBT compared to IPT (23). Unfortunately, the only project comparing CBT with STPP did not investigate the differential impact of cluster A traits (21, 31). One should therefore be careful to draw firm conclusions from the present finding. The amount of variance explained by the interaction term was rather small. Moreover, among the patients with paranoid traits two thirds had only one trait, and few patients in the sample had a full diagnosis of paranoid PD. Even if a low number of paranoid traits may be typical for samples with less severe PD, the small sample size in the present study limits the generalizability of this result, which must be interpreted with cautiousness. It’s also a possibility that the result reflects therapist effects, countertransference reactions, or other therapeutic processes specific to this study. The finding must thus be replicated in future research that includes larger samples. Additionally, valuable knowledge may be obtained from process analyses of patients with depression and complex relational problems.

### 6.1 Strengths and limitations

The present study was part of the MOP project conducted at two outpatient clinics within the public mental health care and has high ecological validity for patients with MDD and mild to moderate PD. Avoidant PD was the most prevalent PD, a common finding in outpatient samples (19, 21, 23, 51, 59). Severe PD was not an exclusion criterion in the MOP project, and the study sample, with less severe PD, probably reflects a clinical practice where more severe disorders or borderline PD are referred to other treatment programs available in the services. The findings may thus not be representative of clinics with more restricted treatment offers, but may illustrate the comparable potential for improvements for depressed patients with mild to moderate PD in settings with opportunities to refer patients to specifically tailored PD treatments. Moreover, the treatments were delivered by well-trained therapists under regular supervision, which is a strength of the study, but could limit generalizability to less resourceful contexts. PD was assessed by semi-structured interviews with high reliability. The study combined a categorical and dimensional approach to personality pathology, and analyses investigating the influence of specific PD traits controlled for the presence of other PD criteria. Yet number of fulfilled SCID-II criteria is a sub-optimal operationalization of severity of personality pathology. ICD-11 and the DSM-5 alternative Model of Personality Disorder conceptualize severity in terms of level of personality functioning (56, 60). Future studies may benefit from dimensional assessment of levels of personality functioning using interviews or self-report scales developed specifically for this purpose (61–63).

Depression was evaluated by both observer rated and self-report measures showing the same trends. The PD group had a higher level of HDRS at baseline, and we cannot exclude the possibility that the greater change in HDRS among PD patients expresses a regression to the mean effect. Baseline severity of depression is often associated with a poorer outcome (12, 18, 54, 64), and we therefore controlled for baseline levels of depression and other outcome measures when investigating the significance of the extent of personality pathology and specific PD traits. Most of the patients had recurrent MDD with equal distribution in the NoPD and PD group. However, age of onset, chronicity, number of previous episodes, or duration of current episode that have been shown to influence the course of depression, although not consistently, were not assessed in the present study (52, 53, 65).

Most reviews and meta-analyses of the influence of PD comorbidity have restricted their focus to depression outcome (8–10, 12, 66), and the inclusion of additional secondary outcomes of psychosocial functioning and anxiety is among the strengths of the study. Global impairment and higher levels of anxiety have been reported as negative risk factors regarding remission and relapse of depression (52, 53, 55). The course of such features during treatment may therefore add valuable information relevant to the longer-term course of depressive symptoms. Future studies might cover an even broader range of functional and personality-related outcomes. Moreover, there is a lack of research investigating how PD affects long-term outcome after short-term psychotherapies and there is an urgent need for follow-up studies (21).

The present results must be interpreted in the context of some important limitations. First and foremost, the current sample size and the small number of patients with PD limited the statistical power to find outcome differences related to PD and specific PD traits. Only large ESs between the NoPD and PD group might be detected with 80% power, and the power to discover moderator effects was low (32). The findings must therefore be replicated in larger samples. Next, despite the large number of statistical tests in the study, which included both primary and secondary outcomes, no attempt was made to control type I errors. An alpha level of p<.05 was applied to balance the risk of type I and type II errors, but we cannot rule out the possibility that for instance the results regarding paranoid traits were random findings reflecting type I errors. Finally, even if the distribution of patients using antidepressants did not differ statistically at baseline or follow-up, neither between the NoPD and PD group nor between treatments, we did not investigate the influence of medication.

## 7 Conclusions

The present study found comparable outcomes in patients with and without PD and indicates that co-occurring mild to moderate personality pathology should not be a barrier to standard psychotherapies for patients with depression. Whether certain types of psychotherapy are more helpful than others regarding personality pathology is still uncertain, and the potential of psychodynamic therapies in comparison with CBT is largely unknown due to a scarcity of studies. The finding that patients with paranoid traits profited more in STPP is noteworthy but needs to be replicated. To move towards an evidence base for personalized treatment, more studies should investigate whether the type and complexity of personality pathology differentially affect short- and longer-term outcomes of different psychotherapies for depressed patients.

## Data Availability

The dataset used and analyzed in this study can be obtained from the corresponding author upon request.

## References

[1] Organization WH . World mental health report: Transforming mental health for all. World Health Organization (2022).

[2] Cuijpers P Karyotaki E Ciharova M Miguel C Noma H Furukawa TA . The effects of psychotherapies for depression on response, remission, reliable change, and deterioration: a meta-analysis. Acta Psychiatr Scand. (2021) 144:288–99. doi: 10.1111/acps.13335 34107050 PMC8457213

[3] Cuijpers P Noma H Karyotaki E Vinkers CH Cipriani A Furukawa TA . A network meta-analysis of the effects of psychotherapies, pharmacotherapies and their combination in the treatment of adult depression. World Psychiatry. (2020) 19:92–107. doi: 10.1002/wps.20701 31922679 PMC6953550

[4] Voderholzer U Barton BB Favreau M Zisler EM Rief W Wilhelm M . Enduring effects of psychotherapy, antidepressants and their combination for depression: a systematic review and meta-analysis. Front Psychiatry. (2024) 15:1415905. doi: 10.3389/fpsyt.2024.1415905 39664326 PMC11632389

[5] Cuijpers P . How to improve outcomes of psychological treatment of depression: lessons from “next-level” meta-analytic research. Am Psychol. (2024) 79:1407. doi: 10.1037/amp0001387 39715395

[6] Friborg O Martinsen EW Martinussen M Kaiser S Øvergård KT Rosenvinge JH . Comorbidity of personality disorders in mood disorders: a meta-analytic review of 122 studies from 1988 to 2010. J Affect Disord. (2014) 152:1–11. doi: 10.1016/j.jad.2013.08.023 24120406

[7] Winsper C Bilgin A Thompson A Marwaha S Chanen AM Singh SP . The prevalence of personality disorders in the community: a global systematic review and meta-analysis. Br J Psychiatry. (2020) 216:69–78. doi: 10.1192/bjp.2019.166 31298170

[8] Newton-Howes G Tyrer P Johnson T . Personality disorder and the outcome of depression: meta-analysis of published studies. Br J Psychiatry. (2006) 188:13–20. doi: 10.1192/bjp.188.1.13 16388064

[9] Newton-Howes G Tyrer P Johnson T Mulder R Kool S Dekker J . Influence of personality on the outcome of treatment in depression: systematic review and meta-analysis. J Pers Disord. (2014) 28:577–93. doi: 10.1521/pedi_2013_27_070 24256103

[10] Young M . Prognostic significance of personality disorders in patients with major depressive disorder. Curr Treat Opt Psychiatry. (2020) 7:559–69. doi: 10.1007/s40501-020-00227-7 41933263

[11] Mulder RT . Personality pathology and treatment outcome in major depression: a review. Am J Psychiatry. (2002) 159:359–71. doi: 10.1176/appi.ajp.159.3.359 11869996

[12] Banyard H Behn AJ Delgadillo J . Personality disorders and their relation to treatment outcomes in cognitive behavioural therapy for depression: a systematic review and meta-analysis. Cogn Ther Res. (2021) 45:561–76. doi: 10.1007/s10608-021-10203-x 41933263

[13] van Bronswijk SC Köster EM Peeters FP . Effectiveness of acute-phase treatment of depression is not influenced by comorbid personality disorders: results from a meta-analysis and meta-regression. Psychother Psychos. (2020) 89:109–10. doi: 10.1159/000502918 31590174 PMC7158226

[14] Kavanagh BE Ashton MM Cowdery SP Dean OM Turner A Berk M . Systematic review and meta-analysis of the role of personality disorder in randomised controlled trials of pharmacological interventions for adults with mood disorders. J Affect Disord. (2021) 279:711–21. doi: 10.1016/j.jad.2020.10.031 33197840

[15] Kool S Schoevers R de Maat S Van R Molenaar P Vink A . Efficacy of pharmacotherapy in depressed patients with and without personality disorders: a systematic review and meta-analysis. J Affect Disord. (2005) 88:269–78. doi: 10.1016/j.jad.2005.05.017 16165217

[16] American Psychiatric Association D American Psychiatric Association D . Diagnostic and statistical manual of mental disorders: DSM-5 Vol. 5. . Washington, DC: American psychiatric association (2013).

[17] WHO . ICD-11 clinical descriptions and diagnostic guidelines for mental and behavioural disorders. In: World Health Organisation. WHO, Geneva (2022). 10.1002/wps.20189 PMC4329901 25655162

[18] Carter JD Luty SE McKenzie JM Mulder RT Frampton CM Joyce PR . Patient predictors of response to cognitive behaviour therapy and interpersonal psychotherapy in a randomised clinical trial for depression. J Affect Disord. (2011) 128:252–61. doi: 10.1016/j.jad.2010.07.002 20674982

[19] Joyce PR McKenzie JM Carter JD Rae AM Luty SE Frampton CM . Temperament, character and personality disorders as predictors of response to interpersonal psychotherapy and cognitive-behavioural therapy for depression. Br J Psychiatry. (2007) 190:503–8. doi: 10.1192/bjp.bp.106.024737 17541110

[20] Ryder AG Quilty LC Vachon DD Bagby RM . Depressive personality and treatment outcome in major depressive disorder. J Pers Disord. (2010) 24:392–404. doi: 10.1521/pedi.2010.24.3.392 20545502

[21] Koppers D Kool M Van H Driessen E Peen J Dekker J . The effect of comorbid personality disorder on depression outcome after short-term psychotherapy in a randomised clinical trial. BJPsych Open. (2019) 5:e61. doi: 10.1192/bjo.2019.47 31530296 PMC6646965

[22] Shea MT Pilkonis PA Beckham E Collins JF Elkin I Sotsky SM . Personality disorders and treatment outcome in the NIMH Treatment of Depression Collaborative Research Program [Clinical Trial Randomized Controlled Trial Research Support, U.S. Gov't, P.H.S. Am J Psychiatry. (1990) 147:711–8. doi: 10.1176/ajp.147.6.711 2343912

[23] van Bronswijk SC Lemmens LH Viechtbauer W Huibers MJ Arntz A Peeters FP . The impact of personality disorder pathology on the effectiveness of cognitive therapy and interpersonal psychotherapy for major depressive disorder. J Affect Disord. (2018) 225:530–8. doi: 10.1016/j.jad.2017.08.043 28866297

[24] Shapiro DA Barkham M Rees A Hardy GE Reynolds S Startup M . Effects of treatment duration and severity of depression on the effectiveness of cognitive-behavioral and psychodynamic-interpersonal psychotherapy. J Consult Clin Psychol. (1994) 62:522. doi: 10.1037//0022-006x.62.3.522 8063978

[25] Hardy GE Barkham M Shapiro DA Stiles WB Rees A Reynolds S . Impact of Cluster C personality disorders on outcomes of contrasting brief psychotherapies for depression. J Consult Clin Psychol. (1995) 63:997. doi: 10.1037/0022-006x.63.6.997 8543722

[26] Elkin I Shea MT Watkins JT Imber SD Sotsky SM Collins JF . National Institute of Mental Health treatment of depression collaborative research program: general effectiveness of treatments. Arch Gen Psychiatry. (1989) 46:971–82. doi: 10.1001/archpsyc.1989.01810110013002 2684085

[27] Barber JP Muenz LR . The role of avoidance and obsessiveness in matching patients to cognitive and interpersonal psychotherapy: empirical findings from the treatment for depression collaborative research program. J Consult Clin Psychol. (1996) 64:951. doi: 10.1037/0022-006x.64.5.951 8916624

[28] McBride C Atkinson L Quilty LC Bagby RM . Attachment as moderator of treatment outcome in major depression: a randomized control trial of interpersonal psychotherapy versus cognitive behavior therapy [Randomized Controlled Trial Research Support, Non-U.S. Gov't. J Consult Clin Psychol. (2006) 74:1041–54. doi: 10.1037/0022-006x.74.6.1041 17154734

[29] Huibers MJ Cohen ZD Lemmens LH Arntz A Peeters FP Cuijpers P . Predicting optimal outcomes in cognitive therapy or interpersonal psychotherapy for depressed individuals using the personalized advantage index approach. PloS One. (2015) 10:e0140771. doi: 10.1371/journal.pone.0140771 26554707 PMC4640504

[30] Malkomsen A Wilberg T Bull-Hansen B Dammen T Evensen JH Hummelen B . Comparative effectiveness of short-term psychodynamic psychotherapy and cognitive behavioral therapy for major depression in psychiatric outpatient clinics: a randomized controlled trial. BMC Psychiatry. (2025) 25:113. doi: 10.1186/s12888-025-06544-6 39934737 PMC11817821

[31] Kikkert MJ Driessen E Peen J Barber JP Bockting C Schalkwijk F . The role of avoidant and obsessive-compulsive personality disorder traits in matching patients with major depression to cognitive behavioral and psychodynamic therapy: a replication study. J Affect Disord. (2016) 205:400–5. doi: 10.1016/j.jad.2016.08.017 27598693

[32] Røssberg J Evensen J Dammen T Wilberg T Klungsøyr O Jones M . Mechanisms of change and heterogeneous treatment effects in psychodynamic and cognitive behavioural therapy for patients with depressive disorder: a randomized controlled trial. BMC Psychol. (2021) 9:1–14. doi: 10.1186/s40359-021-00517-6, PMID: 33482927 PMC7821688

[33] Hamilton M . A rating scale for depression. J Neurol Neurosurg Psychiatry. (1960) 23(1):56–62. doi: 10.1136/jnnp.23.1.56, PMID: 14399272 PMC495331

[34] Beck AT Steer RA Ball R Ranieri W . Comparison of Beck Depression Inventories -IA and -II in psychiatric outpatients [Comparative Study. J Pers Assess. (1996) 67:588–97. doi: 10.1207/s15327752jpa6703_13 8991972

[35] Pedersen G Urnes Ø Hummelen B Wilberg T Kvarstein E . Revised manual for the Global Assessment of Functioning scale. Eur Psychiatry. (2018) 51:16–9. doi: 10.1016/j.eurpsy.2017.12.028 29510296

[36] Mundt JC Marks IM Shear MK Greist JM . The Work and Social Adjustment Scale: a simple measure of impairment in functioning. Br J Psychiatry. (2002) 180:461–4. doi: 10.1192/bjp.180.5.461 11983645

[37] Spitzer R Kroenke K Williams J . Generalized anxiety disorder 7-item (GAD-7) scale. Arch Intern Med. (2006) 166:1092–7. doi: 10.1037/t02591-000 16717171

[38] Beck AT Rush AJ Shaw BF Emery G DeRubeis RJ Hollon SD . Cognitive therapy of depression. Guilford Publications (2024).

[39] Gabbard GO . Long-term psychodynamic psychotherapy: A basic text. American Psychiatric Pub (2017).

[40] Cregeen S . Short-term psychoanalytic psychotherapy for adolescents with depression: A treatment manual. Routledge (2018).

[41] Hilsenroth MJ Blagys MD Ackerman SJ Bonge DR Blais MA . Measuring psychodynamic-interpersonal and cognitive-behavioral techniques: development of the comparative psychotherapy process scale. Psychother: Theory Res Prac Training. (2005) 42:340. doi: 10.1037/t01865-000 41770175

[42] First MB Williams JB Karg RS Spitzer RL . Structured clinical interview for DSM-5 disorders: SCID-5-CV clinician version. Washington, DC: American Psychiatric Association Publishing (2016).

[43] Sheehan DV Lecrubier Y Sheehan KH Amorim P Janavs J Weiller E . The mini-international neuropsychiatric interview (M.I.N.I.): the development and validation of a structured diagnostic psychiatric interview for DSM-IV and ICD-10. J Clin Psychiatry. (1998) 59(Suppl 20):22–33. 9881538

[44] Koo TK Li MY . A guideline of selecting and reporting intraclass correlation coefficients for reliability research. J Chiropr Med. (2016) 15:155–63. doi: 10.1016/j.jcm.2016.02.012 27330520 PMC4913118

[45] Wang YP Gorenstein C . Psychometric properties of the Beck Depression Inventory-II: a comprehensive review. Braz J Psychiatry. (2013) 35:416–31. doi: 10.1590/1516-4446-2012-1048 24402217

[46] Zahra D Qureshi A Henley W Taylor R Quinn C Pooler J . The work and social adjustment scale: reliability, sensitivity and value. Int J Psychiatry Clin Pract. (2014) 18:131–8. doi: 10.3109/13651501.2014.894072 24527886

[47] Johnson SU Ulvenes PG Øktedalen T Hoffart A . Psychometric properties of the general anxiety disorder 7-item (GAD-7) scale in a heterogeneous psychiatric sample. Front Psychol. (2019) 10:1713. doi: 10.3389/fpsyg.2019.01713 31447721 PMC6691128

[48] Rutter LA Brown TA . Psychometric properties of the generalized anxiety disorder scale-7 (GAD-7) in outpatients with anxiety and mood disorders. J Psychopathol Behav Assess. (2017) 39:140–6. doi: 10.1007/s10862-016-9571-9 28260835 PMC5333929

[49] Cohen J . Statistical power analysis for the behavioral sciences. Hillsdale, NJ: Lawrence Erlbaum Associates (1988) p. 20–6.

[50] Kelly BD Nur UA Tyrer P Casey P . Impact of severity of personality disorder on the outcome of depression. Eur Psychiatry. (2009) 24:322–6. doi: 10.1016/j.eurpsy.2008.12.004 19195850

[51] Kuyken W Kurzer N DeRubeis RJ Beck AT Brown GK . Response to cognitive therapy in depression: the role of maladaptive beliefs and personality disorders. J Consult Clin Psychol. (2001) 69:560. doi: 10.1002/9780470713242.ch2 11495185

[52] Buckman JE Underwood A Clarke K Saunders R Hollon S Fearon P . Risk factors for relapse and recurrence of depression in adults and how they operate: a four-phase systematic review and meta-synthesis. Clin Psychol Rev. (2018) 64:13–38. doi: 10.1016/j.cpr.2018.07.005 30075313 PMC6237833

[53] Solmi M Cortese S Vita G De Prisco M Radua J Dragioti E . An umbrella review of candidate predictors of response, remission, recovery, and relapse across mental disorders. Mol Psychiatry. (2023) 28:3671–87. doi: 10.1038/s41380-023-02298-3 37957292 PMC10730397

[54] Tagliaferri SD Han LK Khetan M Nguyen J Markulev C Rice S . Systematic review and meta-analysis: Predictors of relapsing, recurrent, and chronic depression in young people. J Am Acad Child Adolesc Psychiatry. (2026) 65(2):206–30. doi: 10.1016/j.jaac.2025.03.019 40154950

[55] Yang H Wu M Huang W Yu H Teng C Yang H . Psychosocial functioning and its influencing factors in patients with depression post-remission: Implications for assessment and interventions. J Affect Disord. (2024) 367:219–28. doi: 10.1016/j.jad.2024.08.210 39226938

[56] American Psychiatric A . Diagnostic and statistical manual of mental disorders: DSM-5. American Psychiatric Publishing (2013).

[57] Altaweel N Upthegrove R Surtees A Durdurak B Marwaha S . Personality traits as risk factors for relapse or recurrence in major depression: a systematic review. Front Psychiatry. (2023) 14:1176355. doi: 10.3389/fpsyt.2023.1176355 37215669 PMC10196019

[58] Markowitz JC Weissman MM . Interpersonal psychotherapy: principles and applications. World Psychiatry. (2004) 3:136. 16633477 PMC1414693

[59] Kvarstein EH Frøyhaug M Pettersen MS Carlsen S Ekberg A Fjermestad-Noll J . Improvement of personality functioning among people treated within personality disorder mental health services. A longitudinal, observational study. Front Psychiatry. (2023) 14:1163347. doi: 10.3389/fpsyt.2023.1163347 37229394 PMC10203961

[60] ICD W . Clinical descriptions and Diagnostic Guidelines for Mental and Behavioural Disorders. Geneva: World Health Organisation (2022).

[61] Bach B Brown TA Mulder RT Newton-Howes G Simonsen E Sellbom M . Development and initial evaluation of the ICD-11 personality disorder severity scale: PDS-ICD-11. Pers Ment Health. (2021) 15:223–36. doi: 10.1002/pmh.1510 34002530

[62] First MB Skodol AE Bender DS Oldham JM . User's guide for the structured clinical interview for the DSM-5® alternative model for personality disorders (SCID-5-AMPD). American Psychiatric Pub (2017).

[63] Weekers LC Sellbom M Hutsebaut J Simonsen S Bach B . Normative data for the LPFS‐BF 2.0 derived from the Danish general population and relationship with psychosocial impairment. Pers Ment Health. (2023) 17:157–64. doi: 10.1002/pmh.1570 36317556

[64] Luty SE Carter JD McKenzie JM Rae AM Frampton CM Mulder RT . Randomised controlled trial of interpersonal psychotherapy and cognitive–behavioural therapy for depression. Br J Psychiatry. (2007) 190:496–502. doi: 10.1176/foc.8.1.foc110 17541109

[65] Driessen E Smits N Dekker J Peen J Don F Kool S . Differential efficacy of cognitive behavioral therapy and psychodynamic therapy for major depression: a study of prescriptive factors. Psychol Med. (2016) 46:731–44. doi: 10.1017/s0033291715001853 26750445

[66] van Bronswijk SC van Dijk DA van den Boogaard TM Deen ML Ruhé HG Spijker J . Impact of comorbid personality disorders on depression treatment in routine outpatient care. Am J Psychother. (2021) 74:150–6. doi: 10.1176/appi.psychotherapy.20200046 34134502

